# Identifying key covariates of clinical outcomes for critically ill patients with Parkinson’s disease: analysis of the MIMIC-IV database

**DOI:** 10.3389/fneur.2025.1545126

**Published:** 2025-07-03

**Authors:** LiHua Luo, Yang Chen, HangQing Zhao, Rong Dong, Yuzhou Long

**Affiliations:** ^1^Department of Neurology, The Affiliated Hospital of Yunnan University, Kunming, Yunnan, China; ^2^The First People’s Hospital of Yunnan Province, Kunming, Yunnan, China; ^3^School of Clinical Medicine, Dali University, Dali, Yunnan, China

**Keywords:** Parkinson’s disease, MIMIC IV database, covariate, nomogram, prognostic factors, in-hospital mortality

## Abstract

**Background:**

Parkinson’s disease (PD) is a common chronic degenerative disease, and its exact pathological mechanism remains unclear. In this study, we identified covariates associated with the clinical outcomes of PD using patient data from the Medical Information Mart for Intensive Care IV (MIMIC-IV) database, providing new references for the treatment of patients.

**Methods:**

The data of patients with PD and relevant covariates were obtained from the MIMIC-IV database. The patients were categorized into the in-hospital death and in-hospital survival groups based on their survival status, and the relationship between their outcomes and covariates was investigated. Key covariates markedly associated with the clinical outcomes of PD were further screened using regression analysis. Finally, a nomogram for predicting the risk of in-hospital mortality in patients with PD was constructed and validated.

**Results:**

A total of 143 patients with PD and 37 covariates were included in this study. Of the included patients, 25 were assigned to the in-hospital death group and 118 were assigned to the in-hospital survival group. Covariates such as the respiratory rate, mean arterial pressure (MAP), respiratory failure, anion gap, bicarbonate levels, blood urea nitrogen levels, and sequential organ failure assessment (SOFA) scores were markedly associated with in-hospital mortality in patients with PD. Subsequently, age [hazard ratio (HR) = 1.0565, 95% confidence interval (CI) = 1.0065–1.1090, *p* < 0.05], bicarbonate levels (HR = 0.8988, 95% CI = 0.8310–0.9722, *p* < 0.05), BUN levels (HR = 1.0292, 95% CI = 1.0084–1.0503, *p* < 0.05), and SOFA scores (HR = 1.1510, 95% CI = 1.0324–1.2831, *p* < 0.05) were identified as key covariates associated with in-hospital mortality. The nomogram incorporating these covariates exhibited favorable performance in predicting the risk of in-hospital mortality in patients with PD.

**Conclusion:**

This study revealed four key covariates associated with the clinical outcomes of PD, providing new references for the treatment of patients.

## 1 Introduction

Parkinson’s disease (PD) is a chronic, progressive neurodegenerative disorder characterized by the degeneration of dopaminergic (DA) neurons in the substantia nigra pars compacta and the subsequent reduction of striatal dopamine content (1). The loss of DA neurons triggers various motor symptoms, such as tremors, muscle rigidity, bradykinesia, and balance disorders (2). Although PD is defined as a movement disorder, numerous studies have shown that PD causes non-motor symptoms such as cognitive impairment, emotional disturbances, sleep disorders, and autonomic dysfunction in most patients (3). The prevalence of PD is projected to double within the next three decades, making it one of the leading causes of neurodisability (4). While the majority of PD cases are sporadic, approximately 10–15% exhibit familial inheritance patterns linked to mutations in genes such as LRRK2, SNCA, and PARK7. The motor and non-motor symptoms of PD reduce the quality of life and shorten life expectancy, increasing the mortality rate of patients with PD compared with that of the general population (5). Age, gender, and comorbidities have been identified as risk factors of PD (6, 7). The severity of symptoms, timing of symptom onset, and interactions between symptoms vary greatly across patients, making it difficult to establish a uniform prognostic standard (8). In the advanced stages of Parkinson’s disease, patients face an increased risk of complications such as pneumonia, diabetes, and hypertension (9), which often require treatment in the intensive care unit (ICU). Research indicates that individuals with Parkinson’s disease develop these acute conditions more frequently than the general population, leading to a higher likelihood of ICU admissions (10). Furthermore, the dopaminergic medications used to manage Parkinson’s symptoms can interact with commonly administered ICU drugs, such as sedatives, complicating clinical management and potentially worsening patient outcomes (11). Together, these factors contribute to higher mortality rates among Parkinson’s disease patients in the ICU setting. To date, no strategies that can cure or prevent PD have been developed (12). Therefore, identifying novel prognostic factors for PD is necessary, as it may provide a valuable reference for developing more effective treatments.

Medical Information Mart for Intensive Care IV (MIMIC-IV) is a publicly available large-scale medical database developed by the Computer Science Laboratory at Beth Israel Deaconess Medical Center (BIDMC) (13). It provides the health-related data of patients admitted to intensive care units (ICUs), helping medical researchers understand diseases, improve treatments, and develop new therapeutic techniques (14). The breadth and detail of the MIMIC-IV database make it an ideal resource to investigate diseases such as PD, as it contains extensive clinical data that can be used to analyze the epidemiological features, risk factors, and clinical outcomes of diseases (15, 16).

This study focused on sporadic PD cases within the general ICU population. Using the MIMIC-IV database and applying machine learning algorithms such as Least Absolute Shrinkage and Selection Operator (LASSO) regression, the study identified risk factors associated with PD prognosis. The aim was to develop and validate a model for predicting the risk of in-hospital mortality in PD patients, offering new approaches to improve PD prognosis.

## 2 Manuscript formatting

### 2.1 Study design

**Figure 1:**
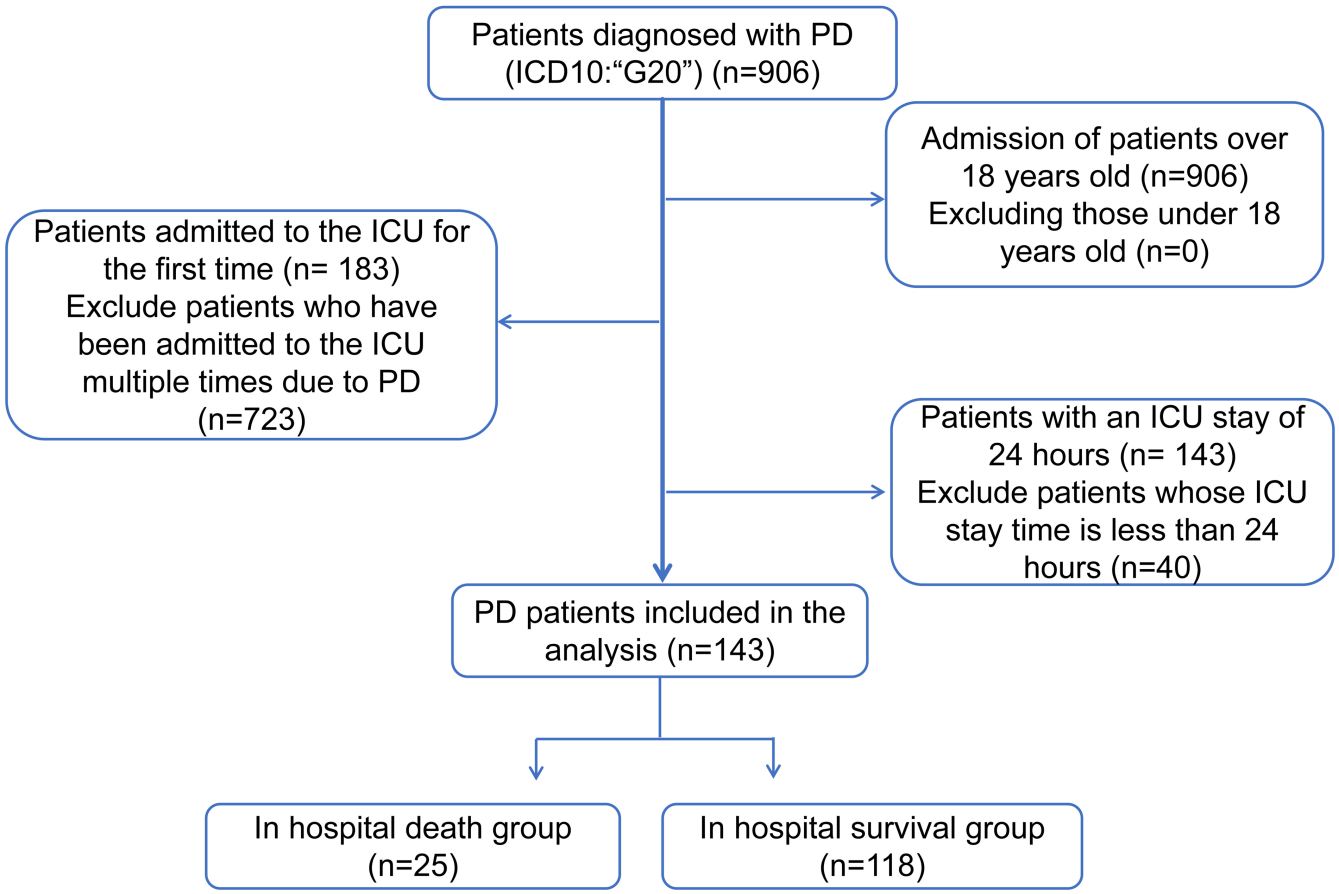
Patient scheduling flowchart.

The MIMIC-IV database^1^ included 906 patients with PD (International Classification of Diseases [ICD10]: “G20”) from 2008 to 2019. The exclusion criteria for this study were as follows: (1) less than 18 years of age at ICU admission, (2) availability of data of only the first ICU admission in patients with multiple ICU admissions, and (3) an ICU stay of less than 24 h. Patients were screened as shown in Figure 1. Eventually, a total of 143 patients (Supplementary Table 1) with PD were included in this study. Patients newly diagnosed with Parkinson’s disease were included in the study. Patient types are shown in Supplementary Table 2, and hospitalization records in Supplementary Table 3. The BIDMC Institutional Review Board waived the requirement for informed consent and approved this study. All data analyzed in this study can be accessed online at https://physionet.org/content/mimiciv/1.0/.

### 2.2 Extraction of covariates and baseline characteristics

Patients with PD were divided into the in-hospital death and in-hospital survival groups based on their survival status. Subsequently, 37 baseline characteristics of these patients were extracted as covariates to investigate the relationship between them and clinical outcomes. The specific definitional values are shown in Table 1. Notably, covariates analysis was conducted using the CreateTableOne function from the R package “tableone” (v 0.13.2).^2^ Continuous variables were presented as mean ± standard deviation. The Shapiro–Wilk normality test was performed for continuous variables. For between-group comparisons, independent samples t-tests (for normally distributed data) and Mann–Whitney U tests (for non-normally distributed data) were performed, with statistical significance set at *p* < 0.05. Categorical variables were expressed as counts and percentages and compared using chi-square tests (*p* < 0.05). To evaluate the influence of covariates on PD patients, eligible cases from the MIMIC database between 2008 and 2019 were selected based on predefined inclusion and exclusion criteria. Patients were divided into two groups based on the in-hospital outcomes: the in-hospital mortality group (*n* = 25) and the in-hospital survival group (*n* = 118). Covariates were analyzed using t-test for continuous variable to identify statistically significant associations with in-hospital mortality (*p* < 0.05). To obtain the overall survival trend of 143 patients, we used the Kaplan-Meier method for survival analysis. Survival time was defined as the time from admission to discharge (event = 1), with the last follow-up time as the censoring value for lost-to-follow-up cases. During data preprocessing, we excluded cases with abnormal survival times (e.g., discharge before admission), variables with over 70% missing rates, and patients with ICU stays shorter than 24 h. In the analysis, the survival curve was fitted using the survfit() function from the R package “survminer,” and the curve was visualized with the ggsurvplot() function.

**Table 1:** Baseline characteristics.

Datatype	Covariates	Level	Type
Demographic data	Race	Black	Categorical variable (%)
		Other	
		White	
	Gender	Female	
		Man	
	Age		Continuous variable [mean (SD)]
Vital signs on the first day of ICU hospitalization	Weight (kg)		
	Temperature (°C)		
	Beats per minute (bpm)		
	Respiratory rate (times/min)		
	spo2 (%)		
	Mean arterial pressure (MAP, mmHg)		
Anamnesis	Hypertension	No	Categorical variable (%)
		Yes	
	Diabetes	No	
		Yes	
	Respiratory failure	No	
		Yes	
	Cirrhosis	No	
		Yes	
	Atrial fibrillation	No	
		Yes	
Assessment within 24 h of admission to the ICU ward	Glasgow coma scale (GCS)		Continuous variable [mean (SD)]
	Sequential organ failure assessment (SOFA)		
Laboratory findings	pH		
	pco2 (mmHg)		
	po2 (mmHg)		
	Baseexcess (mmol/L)		
	Hematocrit (vol%)		
	White blood cell (WBC, k/μL)		
	Platelets (k/μL)		
	Hemoglobin (g/L)		
	Glucose (mg/dL)		
	Calcium (mmol/L)		
	Chloride (mmol/L)		
	Sodium (mEq/L)		
	Potassium (mEq/L)		
	Creatinine (μmol/L)		
	Anion gap (mEq/L)		
	Bicarbonate (mEq/L)		
	Blood urea nitrogen (BUNmg/dL)		
	inr		
	pt (sec)		
	ptt (sec)		
	Antibiotic	No	Categorical variable (%)
		Yes	

### 2.3 Correlation analysis

To identify covariates associated with the prognosis of PD, the survival package (version 0.4.9) (17) was used to perform univariate Cox regression analysis (hazard ratio (HR) ≠ 1, *p* < 0.05). The results were visualized on forest plots using the forestplot package (version 3.1.3) (18). The covariates selected from the univariate analysis were subjected to a proportional hazard (PH) assumption test (*p* > 0.05) to identify candidate covariates associated with prognosis. Finally, the glmnet package (version 4.1.4) (18) was used to perform LASSO regression. The coefficients of the features that were strongly correlated with the disease were moderately shrunk, while the coefficients of the features with weak correlations were shrunk to zero, thus achieving feature selection. The LASSO regression model was obtained through 10-fold cross-validation, when the model error rate was the smallest, the covariates whose variable coefficients were not penalized to 0 were selected as candidate covariates.

### 2.4 Identification of key covariates and construction of a nomogram

Key covariates significantly associated with PD outcomes were identified through multivariate Cox proportional hazards regression analysis using the R package “survival” v 3.7.0 (19), based on variables selected via LASSO regression. Variables with statistically significant associations (HR ≠ 1, *p* < 0.05) were retained. Subsequently, a PH assumption test was conducted on the variables that passed the screening, and *p*-value > 0.05 indicated that these variables met the PH assumption and remained significantly associated with in-hospital mortality. To further evaluate the impact of the identified factors on PD patients, the rms package (version 6.5-0) (20) was used to construct a nomogram incorporating the key covariates for predicting the risk of in-hospital mortality in patients with PD admitted to the ICU. The total score was calculated by converting the regression coefficients of each risk factor into scores and then summing up these scores. Higher total scores corresponded to higher predicted probabilities of in-hospital death. Subsequently, calibration curves and receiver operating characteristic (ROC) curves were plotted using the pROC package (version 1.18.0) (21) to evaluate the predictive performance of the nomogram. A nomogram model was constructed based on the core variables from the multivariate regression analysis. The values of each variable were converted into corresponding scores according to the regression coefficients and summed to obtain a total score, which was then mapped to the predicted probability of in-hospital death. Subsequently, the actual mortality rates in different predicted probability intervals were determined based on the patients’ actual outcomes. Finally, the R package “rms” was employed to plot a calibration curve, with the model-predicted probabilities on the x-axis and the actual observed probabilities of death on the y-axis. Moreover, a calibration curve was generated via 1,000-fold bootstrap resampling to mitigate overfitting.

### 2.5 Statistical analysis

All statistical analyses were performed in R (version 4.2.2), with statistical significance defined as a *p*-value of <0.05. Descriptive statistics for covariates were generated using the CreateTableOne function from the “tableone” R package (v 0.13.2) (see text footnote 2), which provides comprehensive summaries for both continuous and categorical variables. Shapiro–Wilk normality test was used for continuous variables. For between-group comparisons independent samples t-test (for normally distributed data) and Mann–Whitney U test (for non-normally distributed data) were performed, with statistical significance set at *p* < 0.05. Categorical variables were compared using the chi-square test (*p* < 0.05). To identify covariates associated with PD prognosis, univariate Cox regression analyses were performed using the survival package (version 0.4.9) (17), and LASSO regression was performed using the glmnet software package (version 4.1.4) (18). Finally, multivariate Cox proportional hazards regression analyses were performed using the R software package “survival” v 3.7.0 (19). Those variables of interest that were statistically significant (HR ≠ 1, *p* < 0.05) were retained to identify key covariates that were significantly associated with PD outcomes.

## 3 Results

### 3.1 Baseline characteristics

**Figure 2:**
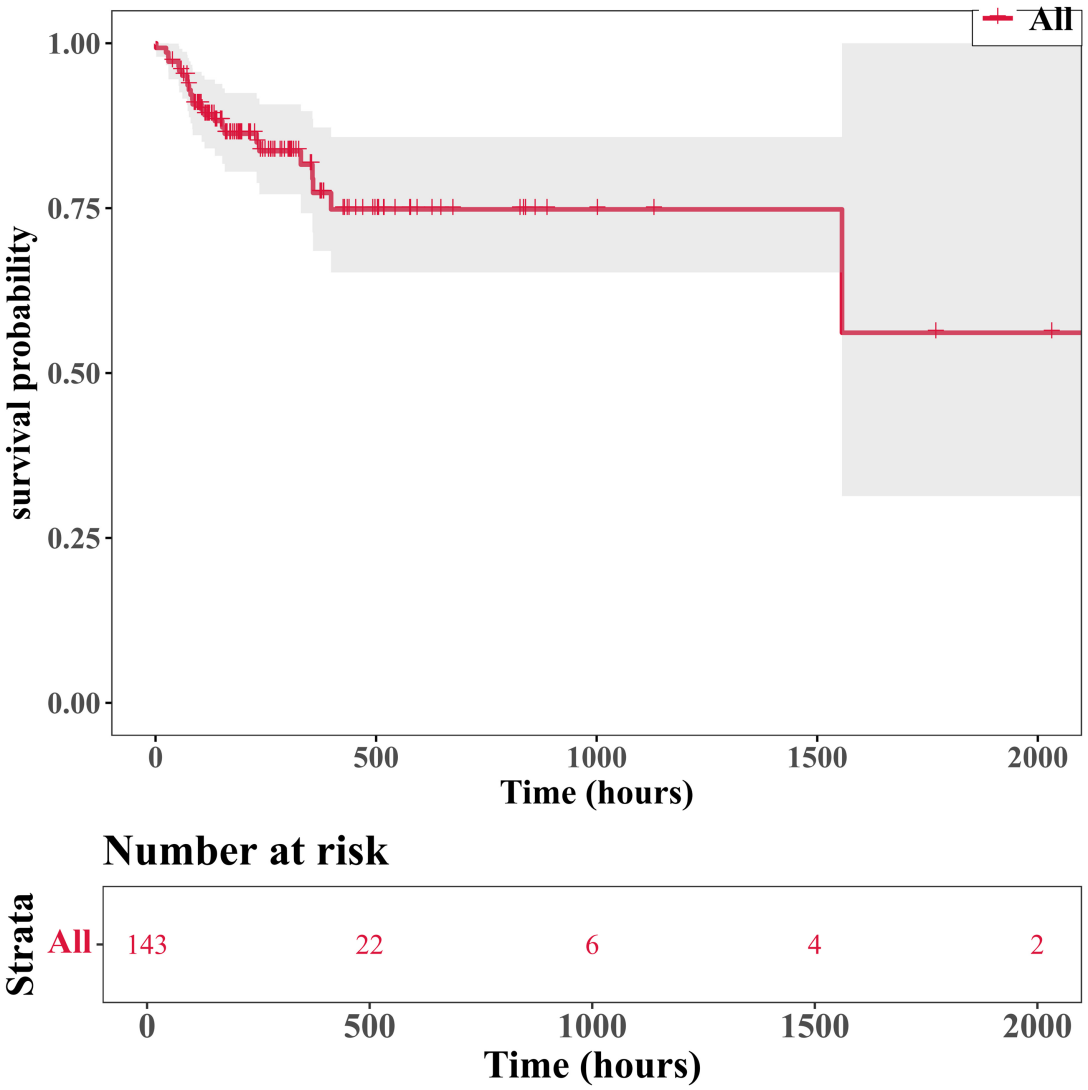
Kaplan–Meier curves of 143 patients.

After analyzing the influence of covariates on patients with Parkinson’s disease, several variables were found to be significantly associated with in-hospital mortality. These included heart rate (beats per minute, *p* = 0.008), mean arterial pressure (*p* = 0.009), respiratory failure (*p* = 0.01), electrolyte imbalance (*p* = 0.017), bicarbonate levels (*p* = 0.005), blood urea nitrogen (*p* = 0.006), and SOFA score (*p* = 0.003) (Table 2). Analysis of the survival status of 143 patients revealed that the survival rate showed a downward trend over time (Figure 2).

**Table 2:** Baseline characteristics of PD patients.

Covariates	Level	In hospital death group	In hospital survival group	*p* value
Race (%)		25	118	
	Black	1 (4.0)	5 (4.2)	0.614
	Other	9 (36.0)	31 (26.3)	
	White	15 (60.0)	82 (69.5)	
Gender (%)	Female	7 (28.0)	40 (33.9)	0.737
	Man	18 (72.0)	78 (66.1)	
Age [mean (SD)]		80.92 (7.87)	77.73 (10.14)	0.141
Weight [mean (SD)]		72.86 (20.95)	72.75 (15.56)	0.977
GCS [mean (SD)]		11.96 (4.31)	12.43 (3.27)	0.537
Temperature [mean (SD)]		36.34 (0.80)	36.39 (0.57)	0.749
Beats per minute (bpm) [mean (SD)]		69.40 (18.68)	67.97 (12.70)	0.642
Respiratory rate [mean (SD)]		10.92 (4.42)	12.92 (3.12)	0.008
spo2 [mean (SD)]		90.52 (7.62)	91.87 (6.97)	0.387
MAP [mean (SD)]		52.76 (19.13)	60.61 (11.90)	0.009
Glucose [mean (SD)]		102.64 (29.33)	104.77 (28.65)	0.737
Hypertension (%)	No	18 (72.0)	69 (58.5)	0.302
	Yes	7 (28.0)	49 (41.5)	
Diabetes (%)	No	18 (72.0)	88 (74.6)	0.987
	Yes	7 (28.0)	30 (25.4)	
Respiratory failure (%)	No	9 (36.0)	78 (66.1)	0.01
	Yes	16 (64.0)	40 (33.9)	
Cirrhosis (%)	No	23 (92.0)	116 (98.3)	0.285
	Yes	2 (8.0)	2 (1.7)	
Atrial fibrillation (%)	No	13 (52.0)	77 (65.3)	0.308
	Yes	12 (48.0)	41 (34.7)	
Hematocrit [mean (SD)]		31.08 (7.20)	31.63 (6.33)	0.705
Hemoglobin [mean (SD)]		9.92 (2.44)	10.30 (2.19)	0.444
Platelets [mean (SD)]		169.72 (75.05)	179.13 (74.29)	0.567
WBC [mean (SD)]		10.18 (4.77)	10.70 (9.43)	0.788
Anion gap [mean (SD)]		14.84 (4.13)	13.07 (3.14)	0.017
Bicarbonate [mean (SD)]		18.24 (4.55)	21.04 (4.50)	0.005
BUN [mean (SD)]		32.28 (18.52)	23.15 (14.02)	0.006
Calcium [mean (SD)]		8.07 (0.83)	8.13 (0.87)	0.741
Chloride [mean (SD)]		104.04 (6.93)	102.53 (6.83)	0.32
Creatinine [mean (SD)]		1.42 (1.01)	1.11 (0.63)	0.052
Sodium [mean (SD)]		139.24 (6.19)	137.83 (7.01)	0.353
Potassium [mean (SD)]		4.02 (0.67)	3.86 (0.53)	0.192
inr [mean (SD)]		1.44 (0.92)	1.25 (0.36)	0.087
pt [mean (SD)]		15.66 (9.76)	13.59 (3.80)	0.078
ptt [mean (SD)]		29.79 (7.53)	30.72 (6.94)	0.549
Antibiotic (%)	No	8 (32.0)	33 (28.0)	0.872
	Yes	17 (68.0)	85 (72.0)	
SOFA [mean (SD)]		7.00 (4.12)	4.85 (3.07)	0.003
po2 [mean (SD)]		104.32 (95.65)	107.01 (95.39)	0.898
pco2 [mean (SD)]		40.88 (11.56)	40.54 (8.65)	0.868
pH [mean (SD)]		7.36 (0.13)	7.38 (0.07)	0.239
Baseexcess [mean (SD)]		−2.48 (5.87)	−0.92 (3.37)	0.073

### 3.2 Identification of four key covariates

**Figure 3:**
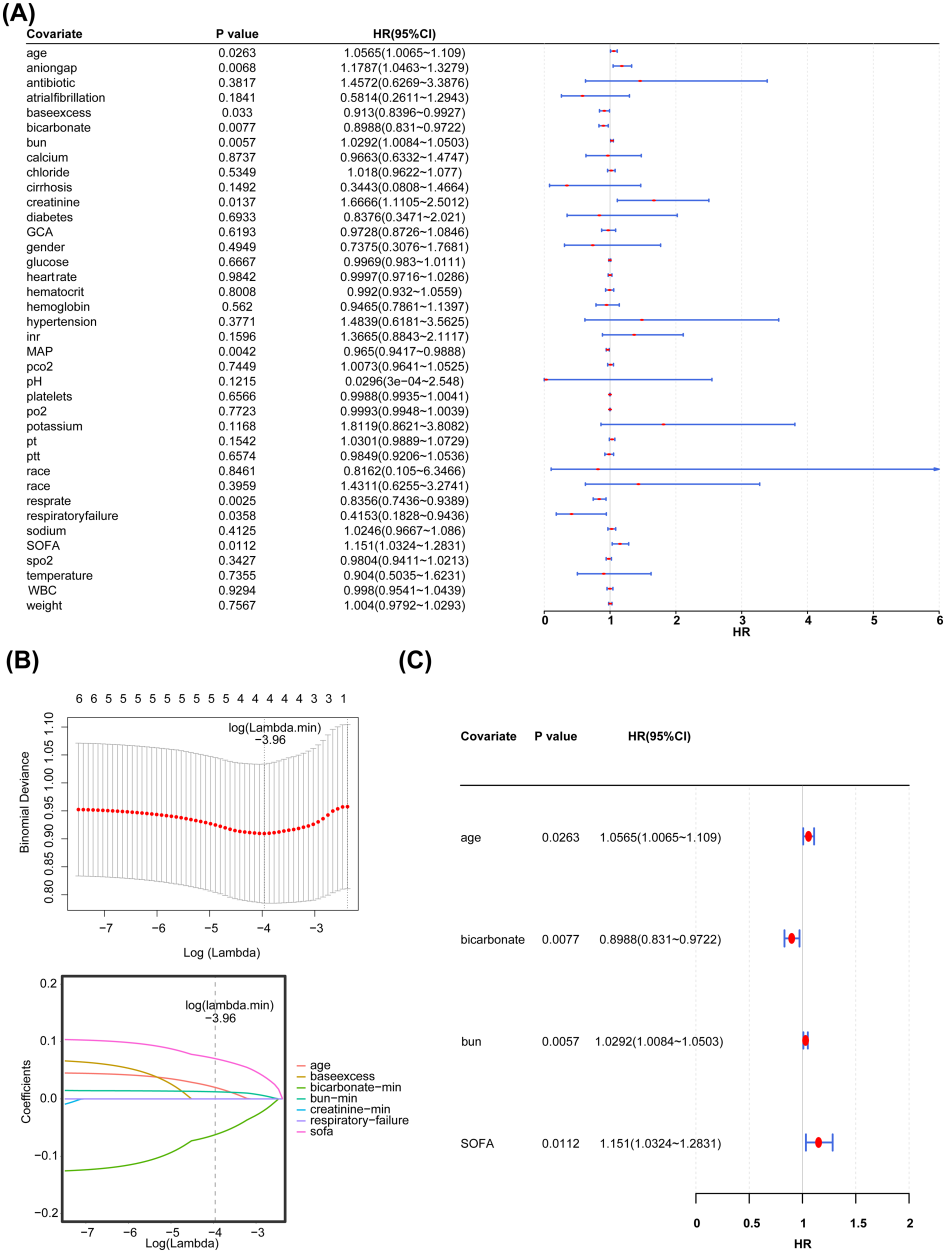
**(A)** Simple linear regression analysis. **(B)** Least absolute shrinkage and selection operator regression analysis (LASSO) regression analysis. The horizontal axis represents the logarithm of lambda, whereas the vertical axis represents the model error. **(C)** Multivariate regression analysis.

Univariate Cox regression analysis showed that age, anion gap, base excess, bicarbonate levels, BUN levels, creatinine levels, MAP, respiratory rate, respiratory failure, and SOFA scores were associated with the prognosis of PD. The PH assumption test (Figure 3A) revealed age, SOFA scores, bicarbonate levels, BUN levels, creatinine levels, Glasgow Coma Scale (GCS) scores, and respiratory failure as candidate covariates associated with the prognosis of PD (Table 3). Further screening via LASSO regression (lambda.min = 0.01906274 [log lambda.min = −3.96]) revealed age, bicarbonate levels, BUN levels, and SOFA scores as candidate covariates (Figure 3B). Finally, multivariate Cox regression analysis and PH assumption test showed that age (HR = 1.0565, 95% confidence interval [CI] = 1.0065–1.1090, *p* < 0.05), bicarbonate levels (HR = 0.8988, 95% CI = 0.8310–0.9722, *p* < 0.05), BUN levels (HR = 1.0292, 95% CI = 1.0084–1.0503, *p* < 0.05), and SOFA scores (HR = 1.1510, 95% CI = 1.0324–1.2831, *p* < 0.05) were key covariates associated with the prognosis of PD (Figure 3C; Table 4). Notably, age, BUN levels, and SOFA scores acted as risk factors for PD (HR > 1).

**Table 3:** PH assumption test of candidate feature covariates.

HR	95% CI		*p* value	PH test *p* value
1.0565	1.0065	1.109	0.0263	0.168231804
0.8988	0.831	0.9722	0.0077	0.059703604
1.0292	1.0084	1.0503	0.0057	0.820351503
1.6666	1.1105	2.5012	0.0137	0.353006849
0.9728	0.8726	1.0846	0.6193	0.987559114
0.4153	0.1828	0.9436	0.0358	0.637139413
1.151	1.0324	1.2831	0.0112	0.098615872

**Table 4:** PH assumption test of feature covariates.

Covariates	HR	95% CI		*p* value	PH test *p* value
Age	1.0565	1.0065	1.109	0.0263	0.168231804
Bicarbonate	0.8988	0.831	0.9722	0.0077	0.059703604
BUN	1.0292	1.0084	1.0503	0.0057	0.820351503
SOFA	1.151	1.0324	1.2831	0.0112	0.098615872

### 3.3 Construction and evaluation of a nomogram

**Figure 4:**
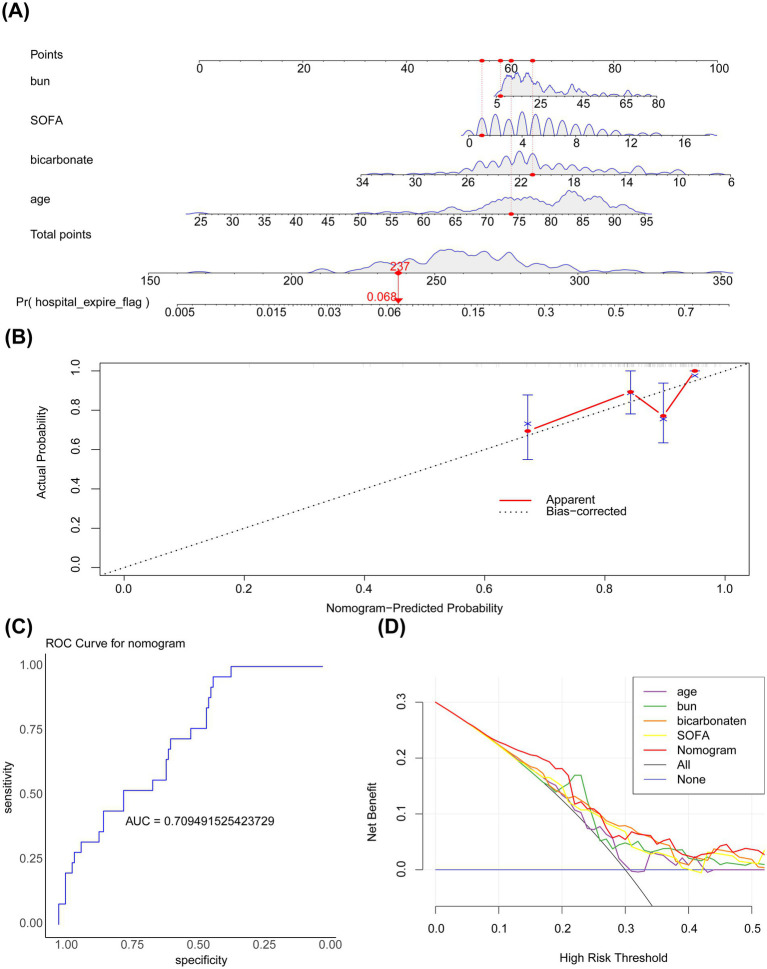
**(A)** Nomogram. The horizontal axis represents the logarithm of lambda, whereas the vertical axis represents the model error. The nomogram is used to predict the risk of in-hospital mortality in patients with PD admitted to the ICU. The bumps in the figure represent subtle variations in the net benefit of model predictions across different threshold probabilities. **(B)** Calibration curve. The horizontal axis represents the predicted event rate, whereas the vertical axis represents the actual event rate. The straight line passing through the origin of the coordinate axes with a slope of 1 serves as the reference line indicating that the predicted probability of the nomogram is exactly the same as the true probability. The closer the predicted value is to the reference line, the more reliable the result is with both ranging from 0 to 1. The ideal prediction corresponds to the black dashed line. **(C)** ROC curve. **(D)** Decision curve analysis. The horizontal axis represents the risk threshold (Pt), whereas the vertical axis represents the net benefit (NB) after subtracting the harm from the benefit. The line segments in the graph represent the net benefit at each risk threshold. The black line parallel to the horizontal axis indicates that all samples are negative, meaning no intervention is applied to any patient. Therefore, the net benefit is 0. The sloping gray line represents the net benefit when all samples are positive, meaning everyone receives the intervention.

A nomogram incorporating the four key covariates was constructed to predict the risk of in-hospital mortality in patients with PD (Figure 4A). Calibration curves demonstrated that the predicted value and the reference line overlapped well (Figure 4B), and the area under the ROC curve (AUC) was 0.709 (Figure 4C). These findings indicated that the nomogram had good accuracy in predicting the risk of in-hospital mortality in patients with PD. Furthermore, DCA showed that the net benefit of the nomogram was >0 and was higher than that of the events wherein all or none of the patients died (Figure 4D). Altogether, the nomogram demonstrated good performance in predicting the risk of in-hospital mortality in patients with PD, representing a new clinical decision-making tool in the management of patients with PD.

## 4 Discussion

PD is a common neurodegenerative disorder characterized by the gradual loss of DA neurons in the substantia nigra, leading to the emergence of motor and non-motor symptoms (1). Given the increasing incidence and mortality rates of PD, the necessity of identifying predictive factors for prognosis is becoming increasingly prominent. In this study, we analyzed the clinical data of patients with PD from the MIMIC-IV database to identify risk factors for in-hospital mortality in patients with PD and construct a predictive model based on these factors, providing a clinical risk assessment and decision-making tool (3). Age, bicarbonate levels, BUN levels, and SOFA scores were identified as key covariates associated with the prognosis of PD.

Age, an important risk factor for PD, was found to be associated with the prognosis of PD in this study. In particular, older patients with PD were found to have a higher risk of in-hospital mortality, a finding consistent with that of previous studies that have emphasized the important role of age in the prognosis of PD (4). Although age is positively associated with the risk of PD, the association between age and in-hospital mortality in patients with PD was not significant. These findings suggest that factors other than age play a crucial role in the progression and prognosis of PD (12).

To the best of our knowledge, this study is the first to demonstrate that blood bicarbonate levels are significantly associated with the risk of in-hospital mortality in patients with PD. This association may be attributed to the role of bicarbonate in regulating blood pH, participating in energy metabolism, and contributing to nervous system function (22). In patients with PD, abnormal bicarbonate levels may reflect metabolic disturbances, which may be closely related to the severity and prognosis of the disease. In patients with metabolic acidosis, serum bicarbonate concentrations are associated with inflammatory responses, such as decreased secretion of IL-10 (23). In addition, changes in the concentration of bicarbonate can affect the concentration of hydrogen ions in the central nervous system, affecting the respiratory rate (24). However, the specific underlying mechanism warrants further investigation.

We found that BUN, an important indicator of renal function, was associated with the prognosis of PD (14). Studies have shown that decreased kidney function, such as a decrease in the estimated glomerular filtration rate (eGFR), is associated with an increased risk of PD (25). In patients with PD, impaired renal function may affect drug metabolism, especially the pharmacokinetics of dopamine agonists, which may lead to improper drug dose adjustment and subsequently influence the therapeutic effect (26). Yang et al. found that BUN levels were significantly higher in patients with PD than in control individuals, suggesting that BUN levels serve as a promising biomarker for PD (27). In addition, elevated BUN levels have been associated with a poor prognosis in patients with acute exacerbation of chronic obstructive pulmonary disease (COPD) (28). Therefore, monitoring and regulating kidney function in patients with PD may help improve their long-term survival.

SOFA score, a tool to assess the extent of organ dysfunction in critically ill patients, was identified as a key covariate associated with the prognosis of PD in this study (15). In particular, patients with PD with higher SOFA scores were at higher risk for in-hospital mortality. This finding highlights the importance of a comprehensive assessment of multiple organ dysfunction in the management of patients with PD (16).

The MIMIC-IV database, a public resource containing the clinical data of patients admitted to ICUs, provides a unique opportunity to explore the characteristics and prognostic factors of diseases, such as PD, in real-world clinical settings (29). Compared with data derived from traditional hospital- or clinic-based studies, the data found in the MIMIC-IV database are comprehensive and diverse and better reflect the overall disease burden and treatment response of patients with PD. In this study, we developed a predictive nomogram incorporating the four key covariates associated with the prognosis of PD. This nomogram showed good accuracy and clinical applicability in predicting the risk of in-hospital mortality in patients with PD, demonstrating the value of using databases such as MIMIC-IV for clinical research.

This study identified four key covariates associated with inpatient mortality in critically ill patients with Parkinson’s disease (PD) by analyzing clinical data from the MIMIC-IV database: age, bicarbonate levels, blood urea nitrogen (BUN) levels, and SOFA scores. These covariates influence the prognosis of PD patients through different biological pathways and clinical processes. Increasing age is associated with a decline in physiological function and a higher prevalence of comorbidities (30), while PD-specific pathophysiological changes further amplify this risk. For example, motor impairments due to neuronal degeneration can compromise airway clearance, increasing the risk of aspiration and pneumonia (31). In addition, dysphagia-related malnutrition and dehydration can exacerbate renal dysfunction (32). Abnormal bicarbonate levels may reflect underlying metabolic disturbances and possible respiratory muscle weakness, which can lead to hypoventilation (33). The SOFA score provides a comprehensive assessment of the severity of multiple organ dysfunction, integrating the impact of these interconnected factors (34). Notably, neurodegeneration in PD patients may alter drug pharmacokinetics, potentially affecting the interpretation of markers such as BUN (35). These findings underscore the importance of implementing personalized management strategies for critically ill PD patients, including early assessment of swallowing function, enhanced airway management, and close monitoring of hemodynamic and metabolic parameters. It should be noted that since non-PD critically ill patients were not included as controls in this study, the mechanistic impacts of these key covariates on PD need to be verified by incorporating controls in future studies.

This study not only introduces a tool for risk assessment of patients with PD in clinical settings but also provides a valuable reference for future research in this field. However, several limitations of this study should be acknowledged. First, owing to the limitations of data sources, the findings of this study should be validated in other populations and clinical settings. Second, this study focused only on hospitalized PD patients and lacked certain clinical indicators, thus limiting our understanding of long-term prognosis and disease progression. The understanding of long-term prognosis and disease progression in these patients remains limited. The database currently lacks comprehensive details regarding the duration and progression of diseases, and there is a limited number of samples that include patients with comorbidities, which has resulted in an absence of thorough analysis concerning their effects. Additionally, the study did not incorporate non-Parkinson’s Disease (PD) critically ill patients as a control group, which could impede our ability to ascertain whether the identified risk factors are unique to PD. This study is constrained by a relatively small sample size and lack of real-world clinical validation for the predictive nomogram, which hinders comprehensive risk factor analysis for in-hospital mortality and compromises the reliability of conclusions. The MIMIC-IV database lacks key information, such as indicators of PD severity, equivalent daily doses of levodopa, and complete records of PD-related comorbidities. These gaps may lead to the omission of PD-specific risk factors or underestimation of interactions between covariates.

To address these limitations, future studies should expand the sample size, include non-PD critically ill patients as a comparison group, and incorporate additional clinical indicators and biomarkers—such as hemodynamic parameters and laboratory values—to enable a more thorough assessment of PD patients’ clinical characteristics. Enhancing the dataset to include complete SOFA scores and multidimensional features of PD is crucial for validating the external applicability of the model. Multicenter prospective studies are also needed to generate high-quality datasets for model refinement. Collaborations with PD-specific databases could further enrich the data by integrating variables such as PD severity, medication regimens, and comorbidities. Multicenter cohort studies should be conducted to address data gaps and clarify the impact of unmeasured factors on ICU outcomes, thereby refining risk prediction models for clinical stratified management. Additionally, long-term follow-up of PD patients is essential to better evaluate disease progression and prognosis, providing clinicians with a stronger evidence base for optimizing care strategies. Furthermore, we plan to include non-PD critically ill patients using MIMIC data for comparative analysis, which will help clarify risk factors that are specific to PD and ultimately offer more valuable insights for clinical decision-making.

Despite the abovementioned limitations, the findings of this study have important clinical implications. Identifying key prognostic factors may facilitate the development of personalized treatment and management strategies for Parkinson’s disease (PD) patients. However, these prognostic mechanisms require further investigation. In addition, this study highlights the potential use of large medical databases in clinical research, providing new methods and tools for future research on PD. With the continuous development of medical information technology, more data resources will be used to develop better healthcare services and improve treatment outcomes for patients with PD.

This retrospective study demonstrated the effects of various clinical characteristics on the clinical outcomes of PD. Initially, patients with PD selected from the MIMIC-IV database were divided into the in-hospital death and survival groups, and various clinical characteristics were compared between the two groups. Subsequently, age, bicarbonate levels, BUN levels, and SOFA scores were identified as four key covariates associated with the prognosis of PD. Finally, a nomogram incorporating these covariates was established to predict the risk of in-hospital mortality in patients with PD. The nomogram showed good predictive accuracy, providing a novel and reliable clinical decision-making tool for the management of patients with PD.

## Data Availability

The original contributions presented in the study are included in the article/Supplementary material, further inquiries can be directed to the corresponding author.

## Footnotes

^1^ https://eye.hms.harvard.edu/bidmc

^2^ https://github.com/kaz-yos/tableone
